# Detection of dental fomites using topical fluorescein

**DOI:** 10.1038/s41415-022-4403-7

**Published:** 2022-07-05

**Authors:** Richard Newsom, Chris Pattison, Adam Amara, Chris Louca

**Affiliations:** 41415124022001 Professor, School of Health and Care Professions, University of Portsmouth, Portsmouth, UK; 41415124022002 Senior Research Associate, Institute of Cosmology and Gravitation, University of Portsmouth, UK; 41415124022003 Director, Institute of Cosmology and Gravitation, University of Portsmouth, UK; 41415124022004 Director and Head of School, University of Portsmouth Dental Academy, Portsmouth, UK

## Abstract

**Background **Thorough disinfection of dental facilities is of paramount importance during the COVID-19 pandemic. Patients, clinicians, students and nurses can all be infected by aerosols and dental droplets bearing COVID-19. However, droplets are transparent and often microscopic, so are difficult to detect in clinical practice.

**Methods** To better understand the spread of dental droplets, we stained the dental irrigant with fluorescein and performed a series of procedures on a dental manikin. We then viewed droplets and fomite spread around the dental chair, with and without an ultraviolet (UV) light.

**Results** Observations without the UV light showed minimal or no fluid spread. However, using UV light, we detected fluorescein on the dentist, chairs and the handpiece, as well as splatter on the floor and on the instrument tray. This was of educational value to the staff, who were reminded how far droplets had spread.

**Conclusion** Fluorescein facilitates the detection of droplet spread and helps clinical staff to see high-risk areas that require in-depth cleaning. As clinical grade fluorescein is cheap and widely available, this technique may be useful for dental practices to train staff in the thorough decontamination of the clinical environment.

## Key points

- Dental droplet spread has been well documented; however, in normal practice, it is undetectable.
- Use of fluorescein enables detection of fomite and droplet spread.
- Thorough cleaning of surfaces reduces fomite spread and the risk of COVID-19 to patients and all dental team members.

## Introduction

COVID-19 spreads through aerosols, droplets and fomites (surface contamination).^1^^,^^2^^,^^3^ The World Health Organisation considers droplet and fomite spread to be important,^4^ as once on a surface, the virus survives for many hours.^5^ Researchers have shown the importance of surface contamination in the spread of COVID-19.^5^ In a previous paper,^6^ we detected 23,000+ microscopic droplets using a 'cough model' and found 5-6% of the target area was covered in droplet fluid.

Within the dental clinic, high speed air turbine dental handpieces and ultrasonic instruments produce considerable quantities of droplets and aerosols. Several studies have shown that electric-driven dental handpieces, using water jet coolant (water only) rather than spray (water and air mix) can reduce this spread.^7^^,^^8^ Current UK dental regulations recommend a 'fallow time' between patients, of 10-60 minutes to reduce the risk of COVID-19 transmission.^9^^,^^10^ There is some uncertainty around this recommendation, as aerosols are difficult to measure within clinical environments and microscopic droplets are typically undetectable.

Fluorescein dye has been used to highlight potential infection transfer during handwashing using glow-gel,^11^ fomites in clinical areas^12^ and splatter during orthodontic debonding processes.^13^

The aim of this paper is to demonstrate how fluorescein can be used to assess potential droplets and fomite spread within a dental clinical and training setting.

We have developed a method of staining aqueous droplets with fluorescein and then using ultraviolent (UV) light illumination and digital photography to detect the droplets.

## Methods

We used a portable dental manikin attached to a dental chair within the University of Portsmouth Dental Academy clinical area. The fluid within the dental unit water bottle was stained with 10 mg/100 ml fluorescein (Bauch and Lomb, UK).

The dentist performed a series of aerosol generating procedures by cutting plastic teeth with a high-speed air turbine dental handpiece in the presence of high-volume aspiration, delivered by a dental nurse.

Following the procedures, we visually observed the surrounding area under normal lighting and then under 30 W UV illumination (Onforu, China) and imaged with a Nikon DC800 camera, 100 mm, F20 lens (Nikon, UK). The study was conducted in accordance with the tenets of the Declaration of Helsinki and under the University of Portsmouth Ethics Committee, being exempt from full submission.

## Results

**Fig. 1:**
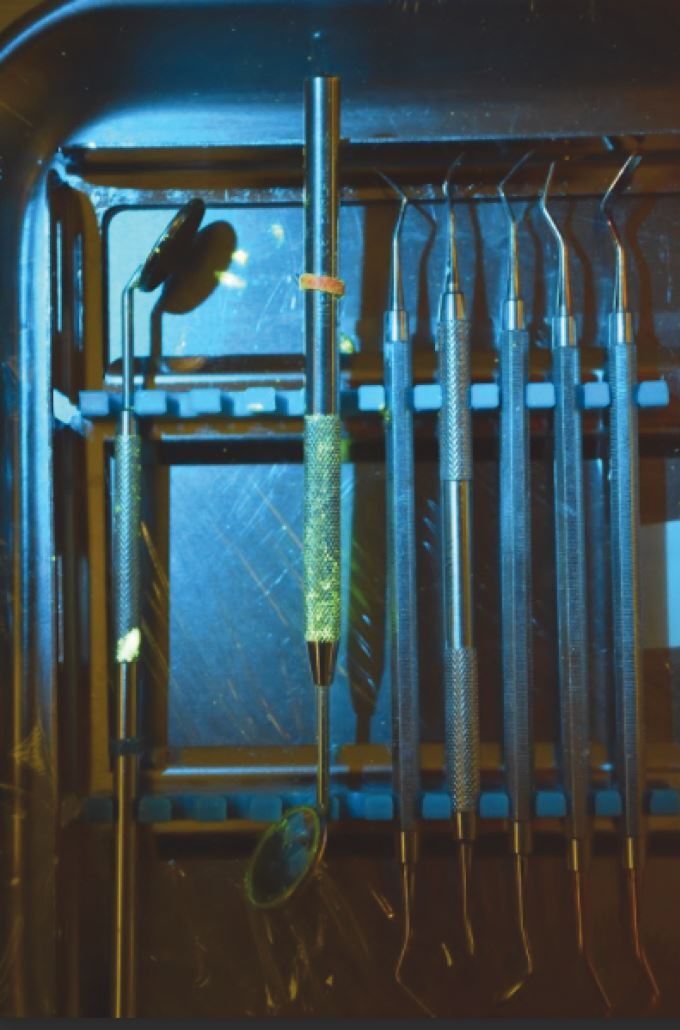
Fluorescein staining of dental instruments. Note that the handle of the mirror is particularly affected

**Fig. 2:**
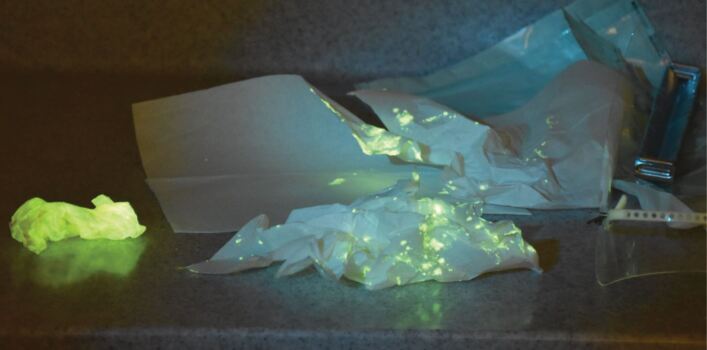
Paper towels and bibs used during the dental procedure. Seeing the amount of fluorescence indicated that they could be highly infective

**Fig. 3:**
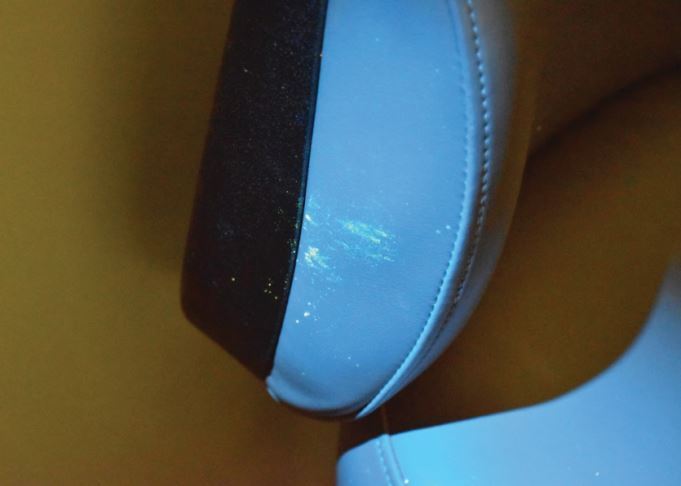
Evidence of fomite transfer of fluorescein on the back and side of the dental chair; these fluorescein markings were in the form of a smear not droplets, indicating they had come from a contaminated hand or instrument

**Fig. 4:**
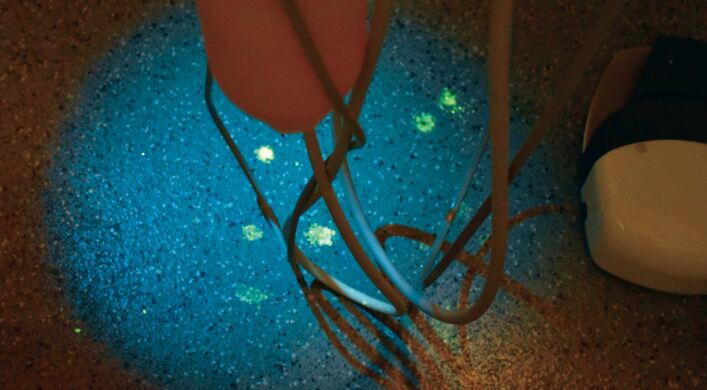
Droplet spread on the floor - these were invisible without UV light

Observations under normal lighting showed minimal or no droplets within the immediate vicinity of the manikin but were visible on its face and teeth. However, under UV light, considerably more droplets and spillages were revealed. We noted fluid splashes on the manikin 'chest', the dental instruments (Fig. 1), on clinical waste (Fig. 2), on the dental chair (Fig. 3) and the clinic floor (Fig. 4). This was of educational value to the clinical staff who were surprised by how far the droplets had travelled.

## Summary

The COVID-19 pandemic has generated a large amount of research on aerosols in dental practice;^14^^,^^9^ it is clear that good ventilation is essential to clear aerosols but measures to locate droplet spread contaminated surfaces are also of importance.

Fluorescein has been previously used to measure the spread of dental water droplets/aerosols around dental chairs.^11^^,^^12^^,^^13^

In our study, using an air turbine and fluorescein-stained irrigant for cutting plastic teeth, it was surprising how far dental fluid had spread and areas for potential fomite transmission were identified. This method could easily be used in clinical practice to determine spread within individual clinics and could be used to train the dental team, highlighting areas that require cleaning between patients.

We have presented a cheap and easy model to replicate droplet spread from a patient that can be used as an education tool in dental practices.

## Conclusion

In conclusion, we have presented a method of detecting droplet splatter using fluorescein and UV light within clinical dental settings. We found it surprisingly easy to miss large areas of fluid contamination without fluorescein staining. This technique can be used for infection control and decontamination training.
